# When social movements fail or succeed: social psychological consequences of a collective action’s outcome

**DOI:** 10.3389/fpsyg.2023.1155950

**Published:** 2023-04-27

**Authors:** Héctor Carvacho, Roberto González, Manuel Cheyre, Carolina Rocha, Marcela Cornejo, Gloria Jiménez-Moya, Jorge Manzi, Catalina Álvarez-Dezerega, Belén Álvarez, Diego Castro, Micaela Varela, Daniel Valdenegro, John Drury, Andrew Livingstone

**Affiliations:** ^1^Escuela de Psicología, Pontificia Universidad Católica de Chile, Santiago, Chile; ^2^School of Psychology and Neuroscience, University of St. Andrews, Scotland, United Kingdom; ^3^School of Psychology, University of Queensland, Brisbane, QLD, Australia; ^4^Department of Social Science, University College London, London, United Kingdom; ^5^Department of Applied Psychology, New York University, New York, NY, United States; ^6^Department of Sociology, University of Oxford, Oxford, United Kingdom; ^7^School of Psychology, University of Sussex, Brighton, United Kingdom; ^8^School of Psychology, University of Exeter, Exeter, United Kingdom

**Keywords:** collective action, social movement, group efficacy, empowerment, social identity

## Abstract

Collective actions occur all around the world and, in the last few years, even more frequently. Previous literature has mainly focused on the antecedents of collective actions, but less attention has been given to the consequences of participating in collective action. Moreover, it is still an open question how the consequences of collective action might differ, depending on whether the actions are perceived to succeed or fail. In two studies we seek to address this gap using innovative experimental studies. In Study 1 (*N* = 368) we manipulated the perceptions of success and failure of a collective action in the context of a real social movement, the Chilean student movement from last decade. In Study 2 (*N* = 169), in addition to manipulating the outcome, we manipulated actual participation, using a mock environmental organization aiming to create awareness in authorities, to test the causal effect of both participation and success/failure on empowerment, group efficacy, and intentions of future involvement in normative and non-normative collective actions. Results show that current and past participation predict overall participation in the future, however, in Study 2 the manipulated participation was associated with having less intentions of participating in the future. In both studies, perception of success increases group efficacy. In Study 1, we found that when facing failure, participants increase their willingness to participate more in the future as opposed to non-participants that actually decrease theirs. In Study 2, however, failure increases the perception of efficacy for those with a history of non-normative participation. Altogether these results highlight the moderating role of the outcome of collective action to understand the effect of participation on future participation. We discuss these results in light of the methodological innovation and the real world setting in which our studies were conducted.

## Introduction

Collective actions are a key aspect towards social change (Thomas and Louis, 2013). From student manifestations against tuition fees in Germany (Tausch et al., 2011), to social protests in Armenia during the Velvet Revolution (Burrows et al., 2022), the Egyptian revolution (Sadowski et al., 2017), and the Chilean social outburst of 2019 (Castro-Abril et al., 2021; Gerber et al., 2023) collective actions have occurred throughout history and all around the world. However, although collective actions are directed towards goals, they rarely achieve them right away (Louis et al., 2020). Moreover, they can have multiple failures and successes through their way to make things change. Therefore, it is not only important to study what mechanisms precede collective actions, but also to understand what happens to those mechanisms when people perceive their participation in collective actions as success or failure. Does it affect intentions of future participation? What reactions may occur? In this paper we go beyond the antecedents of collective actions and focus on what happens after people actually participate in them using an experimental approach rarely seen in collective action research.

### Antecedents of the participation in collective action

In order to achieve their goals, social movements usually mobilize people to collectively carry out actions such as marches, demonstrations, and petitions. The research on this field has mainly focused on identifying antecedents of people’s engagement in collective actions. Perceived injustice, social identity, and the perception that a group is able to accomplish its goals—what has been called group efficacy (Bandura, 2000)—directly affect participation in collective actions, as described in the social identity model of collective action (SIMCA; van Zomeren et al., 2008). Moreover perceived injustice and group efficacy also mediate the effect of social identity on participation.

The encapsulation model of the social identity of collective action (EMSICA; Thomas et al., 2012) and the dual chamber model (Agostini and van Zomeren, 2021) also highlight the importance of social identification, group efficacy and perceived injustice as predictors of participation (Keshavarzi et al., 2021). The dual chamber model and an extended version of SIMCA (van Zomeren, 2013), also add morality as a fourth predictor for collective actions. The extended version of SIMCA also includes group-based anger to account for feelings of injustice.

However, as these models focus on the predictors of participation, they do not account for the consequences that participating in collective actions have on people. Three models that do seek to explain psychological consequences of collective action are the elaborated social identity model (ESIM; Drury and Reicher, 2005, 2009), the collective action recursive empowerment model (CARE; Burrows et al., 2022) and in the dynamic dual pathway model (DDP; van Zomeren et al., 2012; see also Becker and Tausch, 2015 for a different dynamic model that develops SIMCA). ESIM suggests that dimensions of identity can change when participants are socially re-positioned in relation to an outgroup (usually police). In this account, action experienced as successfully imposing ingroup identity over an outgroup leads to psychological empowerment, defined as a positive “social-psychological state of confidence in one’s ability to challenge existing relations of domination” (Drury and Reicher, 2005, p. 35). CARE addresses a dualistic and reiterative process of collective actions that includes individual and group motivations (Burrows et al., 2022). It holds that positive or successful outcomes of small-scale collective actions strengthen motivation for large-scale acts of protest, by increasing feelings of shared group identity, efficacy, and empowerment.

DDP addresses collective action as a dynamic process through a coping perspective in the context of collective disadvantage (van Zomeren et al., 2012). Thus, causes of collective action include a series of cognitive appraisals to cope with disadvantage, such as self-relevance of the collective disadvantage (i.e., group identification), external blame for unfairness, which leads to group-based anger, and group efficacy. Furthermore, participating in collective actions can increase cognitive reappraisals: self-relevance appraisal, the appraisal of unfairness, and the beliefs of being able to cope with the situation, which may lead to increasing group identification.

### Consequences of participating in collective actions

Previous research has shown that when people identifies with a group that is being treated unfairly, they will struggle collectively to improve the group’s social status as it impacts their own sense of wellbeing and self-esteem (Tajfel and Turner, 1979; van Zomeren et al., 2004, 2008). Moreover, participating in collective action can solidify group identity and promote future action (Drury and Reicher, 2005, 2009), it can empower individuals (Drury and Reicher, 2000, 2005) and increase group efficacy beliefs (van Zomeren et al., 2012). Recent studies have also pointed out that after participating in collective action people show higher levels of empowerment (Vestergren et al., 2017; Uluğ and Acar, 2018; Thomas et al., 2022), movement identification, anger (Bilali et al., 2019), and collective effervescence, as shown in a meta-analysis conducted by Pizarro et al. (2022).

A recent review highlighted that participating in collective actions has multiple consequences, including a sustained engagement in collective actions (Thomas et al., 2022). However, there is a distinction within the literature between normative or conventional and non-normative, or radical, collective actions. Normative actions, such as signing petitions, blocking the highway, or participating in peaceful demonstrations, conform to current social norms, while non-normative actions violate these norms, and are often related to more violent methods such as sabotage, or attacks on the police (Tausch et al., 2011).

Lizzio-Wilson et al. (2021) showed that after a failed collective action, people with higher levels of social identification with the unsuccessful group were more likely of both to act in conventional ways and to justify the use of radical methods than those who identify less. Similarly, Louis et al. (2020) found that after a conventional collective action failed, among other reactions, some people more identified with the group were willing to change to more radical tactics. On the contrary, other studies suggest that observers of extreme collective actions identified less with the group, reducing their willingness to join them (Feinberg et al., 2020), and, although participants of radical actions can increase their identification with the movement, if they are against the broader in-group norms, they can disidentify from it (Becker et al., 2011).

Normative and non-violent collective actions promote endorsement of future non-violent strategies by conveying higher perceptions of illegitimacy of the situation and group efficacy (Thomas and Louis, 2014). Likewise, a recent study showed that participation in radical political actions predicted less external political efficacy, i.e., reducing confidence in influencing government decisions; while non-radical political participation predicted higher internal political efficacy, i.e., promoting beliefs of having what is necessary to engage in political activities (Zhu et al., 2022). Therefore, these studies concur with previous results that showed both a positive relation between efficacy and normative actions, and a negative relation between efficacy and non-normative behaviors (Tausch et al., 2011).

### Effects of success and failure

Collective actions are usually goal-oriented. Therefore, outcomes of participation, whether through normative or non-normative actions, are not neutral, and can be evaluated in terms of their success or failure at achieving their goals. However, because of the focus on the predictors of participation in social movements, much less is known about the effect of success or failure of a collective action on the willingness of people to participate further in collective actions. Do people feel more inclined to keep participating once a collective action has succeeded than when it fails?

Previous research has shown that when collective actions succeed, the tactics used tend to be repeated (Louis et al., 2020) and participants are more willing to engage in future collective actions because their participation contributes to increasing group efficacy (van Zomeren et al., 2013). Likewise, Freel and Bilali (2022) suggest that narratives of past successful actions should have a similar effect increasing engagement and group efficacy beliefs. Moreover, feelings of empowerment and motivation for future participation are greater when success is relevant to the social identity of a group (Drury and Reicher, 2009). Meanwhile, a recent review suggests that experiences of collective success in pro environmental actions increase the perception that the group can reach its goals (Fritsche and Masson, 2021). This would be consistent with CARE, where success in small-scale actions increases beliefs of group efficacy and shared identity (Burrows et al., 2022).

Regarding failure, previous research has shown that while it can make people desist at their tasks (Elliott and Dweck, 1988), in a collective context this is not always the case. Sometimes people want to keep on participating in the social movements that have failed to accomplish their goals (e.g., Drury and Reicher, 2000). Also, some field studies report that people can actually unite and feel more empowered when facing failure, leading to more intentions of future participation (Drury et al., 2005; Drury and Reicher, 2005, 2009). This evidence indicates that the effects of success and failure of collective actions are not straightforward.

Moreover, recent studies and a meta-analysis concur that there are divergent and even contradictory responses to failure (Louis et al., 2020, 2022; Lizzio-Wilson et al., 2021). Such responses go from disengagement or disidentification with the group, reducing intentions to act or giving up, to renewed commitment and continued efforts in the movement, which increases intentions to engage. Likewise, it can make people innovate or maintain strategies of conventional or radical actions.

Finally, Tausch and Becker (2013) show that emotions such as pride or anger, related to the success or failure of a movement respectively, enhance motivation for future collective action. Moreover, they suggest that the way the outcome of collective action is interpreted depends on previous level of identification of the participants, such as perceptions of success and failure will have stronger emotional effects on those who identify more with the group. In this study, we aim to investigate if perceptions of success and failure can also moderate the effects of social identity and participation on group efficacy, empowerment, and intentions of future participation.

Thus, previous literature has mainly focused on antecedents of collective action, being social identity, perceived injustice, group efficacy, and empowerment some of the most common variables. Also, more recent studies have investigated consequences of participating in normative or non-normative collective actions, including its effects on the latter variables and future participation. Some of them include perceptions of success or failure as part of the consequences. However, to our knowledge, there is a lack of experimental testing of the outcomes of participating in collective actions. Therefore, in this study we aim to address this gap by experimentally manipulating both participation in collective actions and success or failure, to test whether they have an effect on empowerment, group efficacy and intentions of future involvement in normative and non-normative actions.

### The present studies

In this paper we will present two experimental studies that manipulate the perceptions of success and failure of a social movement in naturalistic environments. In the first study we manipulated the perception of success or failure of a real social movement in Chile known as the student movement. For the second study we created a fictitious pro environmental organization and, in addition to the perception of success or failure, we manipulated the participation in a collective action supposedly carried out by this mock organization. For both studies we sought to maintain the designs as similar as possible, and the statistical analyses conducted for both were exactly the same.

Both experimental studies were carried out in Chile, which has been the scenario of many important social movements over the years, as illustrated by the social movement that brought Salvador Allende to power in 1970, the social movements against Pinochet’s dictatorship in the 80s, the student movements of 2006 and 2011 onwards, and more recently the social outburst of 2019 (Chayinska et al., 2021; Cornejo et al., 2021; González et al., 2021, 2022; Smith et al., 2021; Medel et al., 2022; Gerber et al., 2023). The studies reported here were carried out as part of a broader project addressing the social psychological consequences of participating in collective actions, using longitudinal data (González et al., 2021), mixed methods with dyads of parents and children (Cornejo et al., 2021; González et al., 2021), and, for the case of the studies presented here, experimental data.

Overall, these studies have three main objectives. First, we replicate the previously observed effects of social identity and participation in collective actions on intentions of participation in the future, in both normative and nonnormative collective actions, as well as on perceptions of group efficacy and empowerment. We expect that identifying with a social movement will lead to increased intentions of participation in normative collective actions (Tausch et al., 2011) as well as on feelings of empowerment (Drury and Reicher, 2009) and on the perception that the group is effective (van Zomeren et al., 2004, 2008). Regarding participation we expect that they will have direct effects over intentions of future participation in general (normative and non-normative), but not directly on group efficacy nor empowerment (van Zomeren et al., 2012). All these predictions comprise Hypothesis 1 (*H*_1_).

The second objective of this paper is to determine the effects of success and failure of the social movement over group efficacy, feelings of empowerment and intentions of participating in collective actions in the future. We hypothesize that the perception of success will directly increase perceptions of group efficacy and empowerment (*H*_2_) and indirectly on intentions of future participation through group efficacy (van Zomeren et al., 2012).

Finally, the third objective is to determine the way the perception of success or failure can moderate the effects of social identity and participation (current and past) on group efficacy, empowerment and intentions of future participation. In this case, the evidence is less clear and therefore the hypothesis will remain exploratory (*H*_3_).

## Study 1

During the year 2011, a massive social movement arose in Chile composed mostly of students that demanded free and high-quality college education. With weekly marches and creative demonstrations, the student movement gained much traction and attracted hundreds of thousands of students. As a consequence, the movement widely set the national political agenda for that year. Although with less strength, the movement remained active and organized for a decade, and its effects are still relevant: the government carried out several reforms on the education, tax, and political systems based on the demands of the movement. Whether these outcomes derived from the student movement or were in line with what they sought to accomplish is still a matter of public debate. For this reason, this movement constitutes an appropriate context to manipulate perceptions of success and failure of a social movement and test its psychological effects on its supporters.

### Design

In a 3 × 2 experiment we manipulated the perceptions of either success or failure of the student movement, including a control group; and recruited college students that either participated or not in the actions carried out by the student movement. As previously discussed, one of the main challenges for studying the effects of the success or failure of a social movement is how to precisely define them. To address this, we conducted a pilot study in which we sought to understand how students that identify with the student movement define these outcomes. We conducted semi-structured interviews to students that identify themselves with the movement with different levels of involvement, ranging from non-participants to movement leaders (*N* = 12). We identified two main dimensions in which students assess the success or failure of the student movement: the level of public support and the public expenditure in the education system by the state. Based on these findings, we manipulated the perceptions of success and failure of the movement using a bogus report that stated either the success or the failure of the student movement based on these two dimensions: the levels of public support and public expenditure. In the control condition neither success nor failure was implied by the report. The control condition was included to check whether both experimental conditions, success and failure, work in the expected directions, ruling out that any difference between them might be driven by only one of the conditions working properly.

### Sample and procedures

A team of trained recruiters invited college students in their classrooms to participate in a study that allegedly sought to understand the perceptions of the students regarding the student movement. Each student that completed the questionnaire was offered and paid a retribution of CLP$7000 (approximately US$15). After they consented to participate in the study and accepted to be contacted later via e-mail to answer the questionnaire, they were asked to select one of three phrases that represented them best regarding their involvement with the student movement: *I identify with the student movement and participate in its actions*; *I identify with the student movement but do not participate in its actions*; and *I do not identify with the student movement nor I participate in its actions*. This question allowed us to select participants for the study that identified with the student movement and either participated or not in its actions. We excluded all the students that did not identify with the movement.

The intended sample was of 300 students that were distributed randomly between the success, failure and control conditions, but due to an underestimation of the response rate of the participants, we decided to add a control condition to have a better understanding of the effects of the success and failure. The sample finally consisted of 388 students (*M*_age_ = 20.4, *SD*_age_ = 2.02; 61.3% women). 143 were randomly assigned to the success condition (76 actual participants of the student movement); 147 were randomly assigned to the failure condition (75 actual participants of the student movement) and 78 that were assigned to the control condition (39 actual participants of the student movement). All of them were shown one of the versions of the report. After reading it, they were prompted with a manipulation check, in which they were asked *how successful they though the student movement had been in achieving its objectives* (from 1 = “Not at all successful”; to 5 = “Very successful”); and later proceeded to answer a questionnaire that contained all the dependent variables, as well as questions about demographic information. After completing the questionnaire, all participants were contacted for their monetary retribution and were fully debriefed about the true nature of the study.

### Measures

#### Past participation in collective actions

Past participation in collective actions was measured with an eight-item scale adapted from Tausch et al. (2011), which consisted of two subscales with four items each: Past Participation in Normative Actions of the Student Movement (e.g., ‘Participate in discussion meetings or assemblies’) and Past Participation in Non-Normative Actions of the Student Movement (e.g., ‘Confronting the police in protests’). Participants were asked to rate how often they had participated in those actions in the past year on a scale ranging from 1 (*Never*) to 5 (*Very frequently*). Cronbach’s alpha showed that both sub-scales have good reliability (α_Normative_ = 0.80 and α_Non-normative_ = 0.73).

#### Collective action tendencies

Participation in collective actions was measured with an eight-item scale also adapted from Tausch et al. (2011), which included two subscales with 4 items each: Intentions of Participating in Normative Actions of the Student Movement (e.g., ‘Participate in discussion meetings or assemblies’) and Intentions of Participating in Non-Normative Actions of the Student Movement (e.g., ‘Confronting the police in protests’). Participants were asked to rate how willing they were to participate in actions of the student movement in the future, in a scale ranging from 1 (*Not at all willing*) to 9 (*Very willing*). Cronbach’s alpha showed that both sub-scales have good reliability (α_Normative_ = 0.83 and α_Non-normative_ = 0.82).

#### Social identity

A five-item scale adapted from Leach et al. (2008) was used to assess the students’ level of social identification with the student movement. Some of the items used are *I feel attached to the members of the student movement*; *I am similar to members of the student movement* and *I feel committed to the members of the student movement*. A Likert answer scale ranging from 1 (Strongly disagree) to 5 (Strongly agree) was used. Cronbach’s alpha = 0.86 shows that the scale has good reliability.

#### Empowerment

Two items were used to measure feelings of empowerment: *The student movement is challenging the power of dominant groups in society*; *The student movement has enough power to change social inequality in this country*. A Likert answer scale ranging from 1 (*Strongly disagree*) to 5 (*Strongly agree*) was used. The two items were adequately correlated *r* = 0.52.

#### Group efficacy

A seven-item scale adapted from Tausch et al. (2011) was used to assess the perceptions of group efficacy regarding the Chilean student movement. Some of the items that were used were: *I believe that the Chilean student movement will succeed in implementing reforms in the Chilean educational system;* and *The protests of this movement will be effective to create a change in the Chilean educational system*. A Likert answer scale ranging from 1 (Strongly disagree) to 5 (Strongly agree) was used. Cronbach’s alpha =0.85 shows that the scale has good reliability.

### Results and discussion

To test whether the experimental manipulation worked as expected, a one-way ANOVA was conducted and showed that this was indeed the case, *F*(2,377) = 87.4, *p* < 0.001; participants in the success condition (*M* = 3.82, *SD* = 0.74) perceived that the student movement was more successful than in the control (*M* = 3.18, *SD* = 0.67) and the failure conditions (*M* = 2.74, *SD* = 0.72). A *post-hoc* Tukey test showed that both manipulations differed from the control condition.

All three hypotheses were tested using a general linear model that had the success/failure manipulation (3 conditions) and the current participation quasi experimental conditions (2 conditions) as factors predicting perceptions of group efficacy, feelings of empowerment and intentions of participating in normative and non-normative collective actions of the student movement. Additionally, we included identification with the student movement and reported past participation in the same movement (both normative and non-normative actions) as continuous predictors. This technique is similar to a MANCOVA, with the exception that the latter variables are considered here as independent predictors and not merely as control variables.

*H*_1_: Effects of Social Identity, Current and Past Participation in the Student Movement.

First, we sought to replicate the effects of social identification, current and past participation in a social movement on group efficacy, empowerment, and collective action tendencies. For this, we focused on the main effects of these variables over all dependent variables. As shown in Table 1, social identification with the student movement, *Wilk’s Lambda (V)* = 0.805, *F*(4, 375) = 21.23, *p* < 0.001, *η_p_*^2^ = 0.195; current participation in the movement, *V* = 0.941, *F*(4, 368) = 5.46, *p* < 0.001, *η_p_*^2^ = 0.06; and experiences of past participation in normative, *V* = 0.847, *F*(4, 359) = 15.85, *p* < 0.001, *η_p_*^2^ = 0.15; and non-normative actions of the student movement, *V* = 0.849, *F*(4, 359) = 15.62, *p* < 0.001, *η_p_*^2^ = 0.15; have multivariate effects on the dependent variables.

**Table 1:** Multivariate and univariate main effects of social identity, current participation and past participation in the student movement (normative and non-normative) on perceptions of group efficacy, feelings of empowerment and intentions of participating in collective actions of the student movement.

Independent variable	Dependent variable	*F*	Partial eta squared (observed power)	*B*	*B*’s partial eta squared
Current participation in student movement	Group efficacy	0.052	0 (0)	0.035	0
	Empowerment	2.37	0.007 (0.36)	0.272	0.007
	Collective action tendencies normative	10.356^**^	0.029 (0.91)	−0.096	0
	Collective action tendencies non-normative	13.358^**^	0.036 (0.96)	−0.176	0.001
Social identification with the student movement	Group efficacy	55.304^**^	0.135 (1.0)	0.302^**^	0.041
	Empowerment	35.291^**^	0.091 (0.99)	0.393^**^	0.035
	Collective action tendencies normative	42.396^**^	0.107 (0.99)	0.968^**^	0.08
	Collective action tendencies non-normative	3.025^+^	0.008 (0.40)	0.261	0.004
Past participation in normative actions of the student movement	Group efficacy	5.572^*^	0.016 (0.68)	0.119	0.007
	Empowerment	1.745	0.005 (0.27)	0.126	0.004
	Collective action tendencies normative	62.68^*^	0.151 (1.0)	0.917^**^	0.078
	Collective action tendencies non-normative	11.582^**^	0.032 (0.94)	0.658^**^	0.025
Past participation in non-normative actions of the student movement	Group efficacy	3.924^*^	0.011 (0.52)	−0.207^*^	0.012
	Empowerment	0.078	0 (0)	0.035	0
	Collective action tendencies normative	5.275^*^	0.015 (0.66)	−0.577^*^	0.018
	Collective action tendencies non-normative	39.907^**^	0.102 (0.99)	1.089^*^	0.038
Current participation × Student movement outcome	Group efficacy	0.014	0 (0)		
	Empowerment	0.59	0.003 (0.19)		
	Collective action tendencies normative	2.441^+^	0.014 (0.64)		
	Collective action tendencies non-normative	3.293^*^	0.018 (0.75)		
Social identification × Student movement outcome	Group efficacy	0.987	0.006 (0.33)		
	Empowerment	1.102	0.006 (0.33)		
	Collective action tendencies normative	0.689	0.004 (0.23)		
	Collective action tendencies non-normative	1.297	0.007 (0.37)		
Past participation in normative actions × Student movement outcome	Group efficacy	0.005	0 (0)		
	Empowerment	0.069	0 (0)		
	Collective action tendencies normative	0.222	0.001 (0.09)		
	Collective action tendencies non-normative	2.465^+^	0.014 (0.64)		
Past participation in non-normative actions × Student movement outcome	Group efficacy	1.614	0.009 (0.46)		
	Empowerment	0.104	0.001 (0.09)		
	Collective action tendencies normative	0.49	0.003 (0.19)		
	Collective action tendencies non-normative	2.021	0.011 (0.54)		

^**^p < 0.01; ^*^p < 0.05; ^+^*p* < 0.1. Multivariate effects are shown below each independent variable using Wilk’s Lambda *F* statistic. Within each independent variable, univariate effects on the dependent variables are reported using *F* statistics and Beta values followed by their corresponding effect sizes (partial eta squared). Beta values show the direction of the effect of the independent variable on the dependent variable. Due to interpretation issues, no Beta values are informed for the interactions. Each significant interaction will be interpreted independently.

The univariate main effects of the independent predictors can also be seen in Table 1. Current participation in the student movement (coded 0 = non participant and 1 = participant) had effects on intentions of participating in both normative and non-normative collective actions; *F*(1, 362) = 10.36, *p* = 0.001, *η_p_*^2^ = 0.03, *F*(1, 362) = 36.9, *p* < 0.001, *η_p_*^2^ = 0.04, respectively. In both cases the relation was direct, so that current participants had higher intentions of participating in the future. The same pattern was observed when participants reported having participated in normative collective actions in the past *F*(1, 362) = 62.68, *p* < 0.001, *η_p_*^2^ = 0.15 and *F*(1, 362) = 11.58, *p* < 0.001, *η_p_*^2^ = 0.03 on normative and non-normative actions, respectively. Additionally, we found effects of having participated in normative actions on group efficacy *F*(1, 362) = 5.57, *p* = 0.02, *η_p_*^2^ = 0.02. When students reported having participated in non-normative actions, the pattern was the same: *F*(1, 362) = 5.27, *p* = 0.02, *η_p_*^2^ = 0.02 and *F*(1, 362) = 39.91, *p* < 0.001, *η_p_*^2^ = 0.102 with the same additional effect on group efficacy *F*(1, 362) = 3.94, *p* = 0.048, *η_p_*^2^ = 0.01.

As can be seen in these result patterns, participation consistently predicts more participation; and this pattern seems to be specific to the type of collective action reported. Past participation in normative actions has a stronger effect on intentions of participation in that same type of action, and the same happens when students participate in non-normative actions: they appear to be more willing to be a part of the same types of future actions. Regarding the observed effects on group efficacy, in both cases the results are consistent with previous findings, in that more participation in normative actions predicts more group efficacy, while participation in non-normative actions predicts the opposite pattern, and hence could explain why those students would choose to participate in actions that are explicitly outlawed.

Finally, there were univariate effects of social identification with the student movement on perceptions of group efficacy *F*(1, 362) = 55.3, *p* < 0.001, *η_p_*^2^ = 0.14, empowerment *F*(1, 362) = 35.29, *p* < 0.001, *η_p_*^2^ = 0.09 and intentions of participating in normative collective actions *F*(1, 362) = 42.39, *p* < 0.001, *η_p_*^2^ = 0.11, however not on intentions of participating in non-normative actions *F*(1, 362) = 3.03, *p* = 0.08, *η_p_*^2^ = 0.008. These results are also theoretically consistent given that it has been widely observed that identifying with a group is a strong predictor on each one of these variables.

*H*_2_: Effects of the success of the student movement.

Using the same GLM described before, we tested the main effects of the success/failure experimental manipulation on the dependent variables. We found that there is a marginal multivariate effect of the success of the movement, *Wilk’s Lambda* = 0.963, *F*(8, 700) = 2.71, *p* = 0.1, *η_p_*^2^ = 0.019 over the dependent variables. Although, when inspecting the univariate relations (see Table 2) we found only a significant effect on perceived group efficacy, *F*(2, 374) = 3.28, *p* = 0.039, *η_p_*^2^ = 0.018. The outcome of the social movement did not have an effect on feelings of empowerment, *F*(2,374) = 2.04, *p* = 0.13, *η_p_*^2^ = 0.011; nor on intentions of participating in normative collective actions, *F*(2, 374) = 0.38, *p* = 0.68, *η_p_*^2^ = 0.002 and in non-normative actions, *F*(2, 374) = 0.37, *p* = 0.68, *η_p_*^2^ = 0.002. As can be seen in Table 2, for participants in the success condition, their perception that the social movement was effective in accomplishing its goals was significantly higher than the control and failure conditions. This is consistent with what was theoretically expected from the DDP model, given that when the members of the social movement re-appraise the movement’s ability to cope with their demands, whether they participate or not, they use all the information possible. Therefore, to know that a movement is being successful should have a direct impact on that person’s impression that the movement indeed has the coping capabilities that the situation requires.

**Table 2:** Univariate effects of the success and failure of the student movement on the dependent variables of the model.

	Univariate effects			
	*F*	Partial eta squared (observed power)	Estimated marginal means on experimental conditions	
Group efficacy	3.27^*^	0.018 (0.64)	Success	4.05
			Control	3.79
			Failure	3.73
Empowerment	2.04	0.011 (0.43)	Success	3.50
			Control	3.47
			Failure	3.27
Intentions of participating in normative actions	0.381	0.002 (0.11)	Success	6.33
			Control	6.05
			Failure	6.05
Intentions of participating in non-normative actions	0.378	0.002 (0.11)	Success	3.05
			Control	2.93
			Failure	3.00

^*^p < 0.05. Effects of the experimental manipulation on each of the dependent variables are shown. The effect size of each relation is shown through Partial Eta Squared. Estimated marginal means on each of the experimental conditions are shown for all the dependent variables.

*H*_3_: Moderation effect of the social movement’s outcome.

Finally, to test if the outcome of the social movement has a different effect for the subjects considering their different degrees of current and past participation, as well as their differences in social identification, we estimated four interactions between the success/failure manipulation and each of the continuous predictors, including the quasi experimental factor. Overall, we did not find multivariate effects on neither of the interactions that were estimated, however, there are two interactions that have univariate effects on some dependent variables (see Table 1).

**Figure 1:**
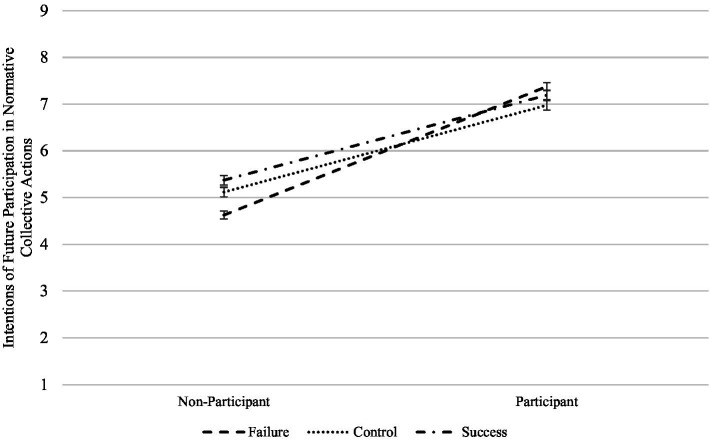
Interaction of social movement success with reported current participation on intentions of participating in normative collective actions.

**Figure 2:**
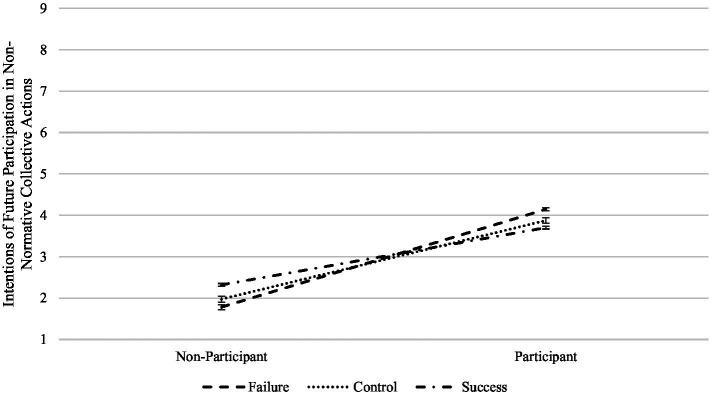
Interaction of social movement success with reported participation on intentions of participating in non-normative collective actions.

**Figure 3:**
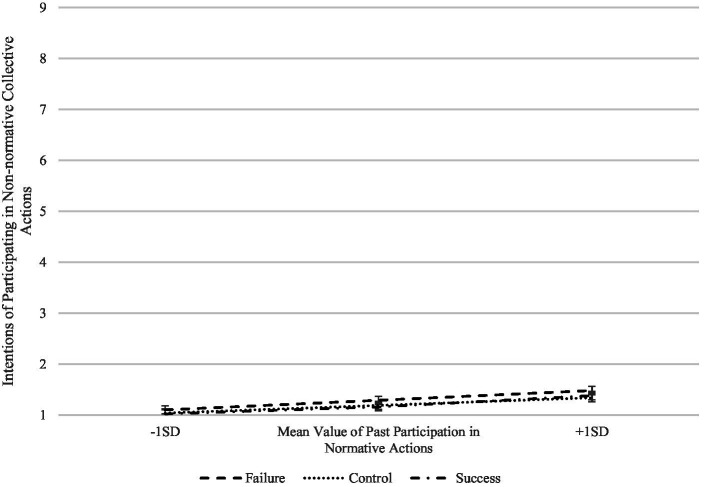
Interaction of social movement success with past participation in normative actions on intentions of participating in non-normative collective actions.

In the first case, the perceived success of the student movement moderates the effect of reported current participation of the students (quasi experimental condition) over their intentions of participating in both normative and non-normative actions, although in the former the effect is only marginally significant, *F*(2,375) = 2.44, *p* = 0.089, *η_p_*^2^ = 0.014 and *F*(2,375) = 3.29, *p* = 0.038, *η_p_*^2^ = 0.018, respectively. As shown in Figure 1, the first interaction shows that when the students are in the failure condition and currently participating in the student movement, their intentions of participating in normative actions of the student movement increase relative to the control and success conditions. The opposite pattern is observed when the students are in the same failure condition but do not participate, that is, they have lower intentions of participating in normative collective actions of the student movement. The second interaction has a similar pattern (see Figure 2). In this case, the students that currently participate in the student movement have higher intentions of participating in non-normative collective actions when facing failure relative to when they perceive success, but not when they do not participate. We found a third interaction in which the student movement’s outcome marginally moderated the effect of past participation in normative actions on intentions of participating in non-normative actions, *F*(2,375) = 2.46, *p* = 0.086, *η_p_*^2^ = 0.014 (see Figure 3). This interaction has a very similar pattern to the previous ones, in that more previous participation predicts more intentions of participating in the future when facing failure, but not when there is no history of participation. Altogether, these results suggest that the outcome of the social movement has a very different effect when the subjects either participate or have a history of participation, especially when facing failure. The fact that social movements fail to accomplish their goals can actually stimulate further participation on already participating individuals.

## Study 2

For this study, we wanted not only to explore the role of the success and failure of a social movement, but also better understand its relationship with actual participation in a collective action. To test this experimentally and have full control over the person’s participation in collective action, we created a bogus organization to experimentally induce a random half of the participants to actually take part in a collective action, leaving the other half as bystanders. To ensure that everyone could identify easily with the organization’s goals we opted for a widespread issue for the population that lives in Santiago: reducing air pollution.

### Design

This experiment had a 2 × 2 design, in which we manipulated the perceptions of success and failure of a social movement, as in Study 1, not including a control condition as in Study 1 both experimental conditions worked properly, and the actual participation in it. To accomplish this, we created a bogus pro-environmental organization that sought to reduce the levels of air pollution in the city. To carry out the experiment, we set two tents in different places of a university campus in Santiago, Chile, and invited potential participants to be informed about the organization’s alleged latest environmental awareness campaign. Additionally, we told the potential participants that the environmental organization partnered with the school of psychology to invite students to take part in a study about the public impact of the campaign, and we could only inform students about the campaign if they agreed to take part in that study. The students that agreed to take part in the study were asked to sign an informed consent and then to go into the tent. Once inside the tent, a research assistant that posed as a member of the organization informed participants individually about the campaign and gave them a brochure that explained the importance of reducing air pollution, as well as the specific goals of the campaign: to inform a specific number of students across universities and to set up an agenda with the environmental authorities to address the issue of air pollution. After this stage, we asked all participants to rate how identified they felt with the organization using a social identity scale. For the manipulation of participation in collective action, a random half of the participants in the experiment was asked to record an audio message that was going to be used to create a political ad for the campaign. The message they had to read was written in a small piece of paper, inside of a bowl full of papers with that same message; although students were led to believe that the messages were different, with the objective of controlling for a potential effect of the message itself. The message consisted of a simple slogan issuing a call to increase environmental awareness. The other half of the students were only informed about the campaign and were not asked to read any message (to be sure, the alleged recorder and bowl of papers were hidden in this condition). One week later, the participants of the study were contacted via email to read a brief report on the results of the organization’s campaign, and then to answer a questionnaire that included the dependent variables and other relevant variables, as well as demographic information. In a random half of the cases, the organization reported that the campaign had been a success, not only because they had informed many more students that they had considered originally, but also because they were able to set a working table with the environmental authorities. In the other half, the report argued that the campaign had been a failure, and the authorities had not agreed to hear the issues that the organization was exposing. After answering the questionnaire, all participants were contacted one more time to hand them their monetary retribution of CLP$11.000 (approximately US$20). In that instance, all of them were thoroughly debriefed about the true nature of the study. Finally, all participants of the study were informed about an actual environmental organization within the university and were given their contact information in case they were interested in participating in an organization that pursued similar goals.

### Sample and procedures

The intended sample was 200 college students, but due to the logistic complexity of the experiment and the restricted time we had in campus to install the tents, the final sample consisted of 169 college students (*M*_age_ = 20.7, *SD*_age_ = 1.95; 51.5% women). 84 students were randomly assigned to the condition of participation (46 of which were assigned to the success condition) and 85 were assigned to the no-participation condition (of which 45 were assigned to the success condition).

### Measures

The same outcome measures were used as in Study 1, although with a few adaptations. Since we were not working with the student movement, the measures of empowerment (*r* = 0.30), group efficacy (α = 0.89) were adapted to fit the context of the bogus organization we created, whereas the measures of collective action tendencies (*α*_Normative_ = 0.82; *α*_Non-normative_ = 0.90) and reported past participation in collective actions (*α*_Normative_ = 0.78; *α*_Non-normative_ = 0.84) were phrased regarding “social causes of your interest,” without specifying a social movement in particular. In the case of social identity, we not only used an adapted measure (*α* = 0.91), but also measured it when the participants were inside the tent before the experimental manipulation of participation, to avoid a potential confound effect.

### Results and discussion

To check the manipulation of participation, at the beginning of the questionnaire we asked participants if they had recorded an audio message in support of the campaign (83 said they did). We cross tabulated their responses with the actual number of participants that were asked to record the message (84 overall) and calculated a Chi-square test *X^2^* = 153.46, *p* < 0.001 that confirmed that the manipulation indeed had taken place. Next, we asked the students to rate how successful they thought the environmental organization had been in accomplishing its goals (from 1 = “Not at all successful”; to 5 = “Very successful”) to check if the manipulation of success and failure had worked as predicted. We conducted an independent samples *t*-test between the conditions which showed that indeed there are statistically significant differences, *t*(167) = −12.64, *p* < 0.001, being that the people in the success condition (*M* = 4.21, *SD* = 0.78) perceive that the environmental organization was being more successful than in the failure condition (*M* = 2.62, *SD* = 0.86).

For the data analysis we conducted exactly the same General Linear Model as in Study 1. The only things that changed were that for this study we only have two conditions for the social movement’s outcome manipulation (success and failure) and the current participation of the subjects was actually manipulated, although the coding remained the same. Both the independent continuous predictors and the dependent variables remained the same. All results are presented in Table 3.

**Table 3:** Multivariate and univariate main effects of social identity, experimental manipulation of participation and past participation in the student movement (H1) and of the experimental manipulation of success and failure (H2) on perceptions of group efficacy, feelings of empowerment and intentions of participating in collective actions of the environmental organization.

	H_1_	Univariate effects			
		*F*	Partial eta squared (observed power)	*B*	B partial eta squared
Experimental manipulation of participation	Group efficacy	5.905^*^	0.036 (0.70)	−0.211^+^	0.02
	Empowerment	5.984^*^	0.036 (0.70)	−0.373^+^	0.03
	Intentions of participating in normative collective actions	4.376^*^	0.027 (0.58)	0.383	0.007
	Intentions of participating in non-normative collective actions	0.135	0.001 (0.07)	−0.092	0
Social identification with organization	Group efficacy	116.686^**^	0.423 (1.0)	0.611^**^	0.238
	Empowerment	34.731^**^	0.179 (0.99)	0.444^**^	0.073
	Intentions of participating in normative collective actions	11.722^**^	0.069 (0.94)	0.452^+^	0.018
	Intentions of participating in non-normative collective actions	0.308	0.002 (0.09)	−0.033	0
Past participation in normative collective actions	Group efficacy	0.197	0.001 (0.07)	0.06	0.004
	Empowerment	0.302	0.002 (0.09)	0.053	0.002
	Intentions of participating in normative collective actions	49.239^**^	0.236 (1.0)	1.231^**^	0.155
	Intentions of participating in non-normative collective actions	9.693^**^	0.057 (0.89)	0.339	0.014
Past participation in non-normative collective actions	Group efficacy	0.199	0.001 (0.07)	−0.161^+^	0.017
	Empowerment	0.525	0.003 (0.11)	0.158	0.008
	Intentions of participating in normative collective actions	2.118	0.013 (0.32)	−0.488	0.017
	Intentions of participating in non-normative collective actions	62.361^**^	0.282 (1.0)	1.822^**^	0.198
	H_2_	*F*	Eta squared	*B*	B eta squared
Experimental manipulation of success and failure	Group efficacy	2.406	0.015 (0.36)	−0.737	0.015
	Empowerment	0.448	0.003 (0.11)	−0.528	0.004
	Intentions of participating in normative collective actions	0.798	0.005 (0.15)	−1.456	0.006
	Intentions of participating in non-normative collective actions	0.34	0.002 (0.09)	−0.826	0.002
	H3	*F*	Eta squared		
Experimental manipulation of participation × Social movement outcome	Group efficacy	0.003	0 (0)		
	Empowerment	0.343	0.002 (0.09)		
	Intentions of participation in normative collective actions	0.39	0.002 (0.09)		
	Intentions of participation in non-normative collective actions	0	0 (0)		
Social identification × Social movement outcome	Group efficacy	1.13	0.007 (0.19)		
	Empowerment	1.009	0.006 (0.17)		
	Intentions of participation in normative collective actions	1.158	0.007 (0.19)		
	Intentions of participation in non-normative collective actions	0.531	0.003 (0.11)		
Past participation in normative collective actions × Social movement outcome	Group efficacy	0.537	0.003 (0.11)		
	Empowerment	0.029	0 (0)		
	Intentions of participation in normative collective actions	0.659	0.004 (0.13)		
	Intentions of participation in non-normative collective actions	0.837	0.005 (0.15)		
Past participation in non-normative collective actions × Social movement outcome	Group efficacy	3.53^+^	0.022 (0.49)		
	Empowerment	0.724	0.005 (0.15)		
	Intentions of participation in normative collective actions	0.698	0.004 (0.13)		
	Intentions of participation in non-normative collective actions	0.662	0.004 (0.13)		

^**^p < 0.01; ^*^p < 0.05; ^+^*p* < 0.1. For each univariate effect, both the F statistic and Beta values are shown with their respective effect sizes (partial eta squared). Beta values show the direction of the effect of the independent variable on the dependent variables.

*H*_1_: Effects of social identity, current and past participation in the student movement.

As in Study 1, we begin reporting the main effects of the continuous predictors. We found multivariate effects of social identification with the environmental organization, *V* = 0.538, *F*(4, 156) = 33.54, *p* < 0.001, *η_p_*^2^ = 0.46; participation in the organization (0 = No Participation Condition; 1 = Participation Condition), *V* = 0.918, *F*(4, 156) = 3.46, *p* = 0.01, *η_p_*^2^ = 0.08; and experiences of past participation in normative *V* = 0.753, *F*(4, 156) = 12.81, *p* < 0.001, *η_p_*^2^ = 0.25 and non-normative actions in social movements in general *V* = 0.662, *F*(4, 156) = 19.89, *p* < 0.001, *η_p_*^2^ = 0.34 on the dependent variables. Table 3 shows the specific univariate effects of the four models.

Exploring the univariate effects of each independent predictor, we found that the experimental manipulation of participating in the bogus organization has a significant main effect over group efficacy *F*(1, 167) = 5.91, *p* = 0.016, *η_p_*^2^ = 0.036 and empowerment *F*(1, 167) = 5.98, *p* = 0.016, *η_p_*^2^ = 0.036. Both effects are in the expected direction, so that participants have higher levels of group efficacy and empowerment; although they did not appear in Study 1, in which the current participation variable only had effects on intentions of future participation. In that regard, the manipulation of participation we did in this study indeed has an effect on the students’ intentions of participating in normative actions in the future, *F*(1, 167) = 6.44, *p* = 0.012, *η_p_*^2^ = 0.038, albeit in an unexpected direction. Students in the participation condition showed significantly lower intentions of future participation (*M* = 5.68) than students in the non-participation condition (*M* = 6.46). This result is contrary to what we found in Study 1, according to which participants had higher intentions of remaining as such relative to non-participants. While this is contradictory, there is evidence that suggests that certain types of low-threshold actions can have this effect on intentions of future participation (see Wilkins et al., 2019).

Mirroring the results of Study 1, social identification with the organization has significant direct effects over perceptions of group efficacy, *F*(1, 167) = 116.69, *p* < 0.001, *η_p_*^2^ = 0.423; feelings of group empowerment, *F*(1, 167) = 34.73, *p* < 0.001, *η_p_*^2^ = 0.179; and intentions of participating in normative actions in the future, *F*(1, 167) = 11.72, *p* = 0.001, *η_p_*^2^ = 0.07; and not over intentions of participating in non-normative collective actions, *F*(1, 167) = 0.31, *p* = 0.58, *η_p_*^2^ = 0.002.

Reported past participation in normative actions of social movements of interest has significant effects over both normative and non-normative collective actions, *F*(1, 167) = 49.24, *p* < 0.001, *η_p_*^2^ = 0.24 and *F*(1, 167) = 9.69, *p* = 0.002, *η_p_*^2^ = 0.057, respectively. Similarly, reported past participation in non-normative actions had an effect over intentions of participating in the same type of actions *F*(1, 167) = 62.36, *p* < 0.001, *η_p_*^2^ = 0.282, but not over normative collective actions *F*(1, 167) = 2.12, *p* = 0.148, *η_p_*^2^ = 0.013. For both participation predictors the direction is as expected, in that more experiences of participation predict future participation, same as in Study 1.

*H*_2_: Effects of the success of the student movement.

Using the same analysis, we tested the main effects of the perceptions of success or failure on each of the dependent variables. Multivariate results indicate that there is no effect of the success of the social movement in the dependent variables, *V* = 0.979, *F*(4, 156) = 0.84, *p* = 0.49, *η_p_*^2^ = 0.02. Univariate results confirm this trend, in that the social movement’s outcome did not have any significant effects over the dependent variables. However, there is a similar pattern over group efficacy to what was observed in Study 1, *F*(1,156) = 2.41, *p* = 0.123, *η_p_*^2^ = 0.015. Even though in this case the result is not significant, effect sizes are practically the same (*η_p_*^2^ = 0.018 for Study 1, and *η_p_*^2^ = 0.015 in Study 2). Whether this is a power issue or actual differences remain to be addressed.

*H*_3_: Moderation effect of the social movement’s outcome.

**Figure 4:**
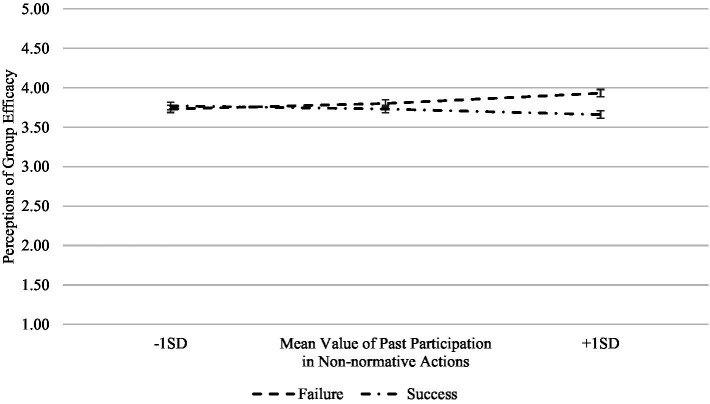
Interaction between success/failure experimental manipulation and past participation in non-normative collective actions on perceptions of group efficacy.

Finally, we tested with the same analysis if the experimental manipulation of success/failure moderated the effects of the independent variables over the dependent variables and found that none of them had multivariate effects (see Table 3). However, we did find that the interaction between history of participation in non-normative actions and the experimental manipulation of the social movement’s outcome have an effect over perceptions of group efficacy *F*(1, 156) = 3.53, *p* = 0.06, *η_p_*^2^ = 0.022 (Figure 4). For people with a history of participation in non-normative collective actions, facing failure actually increases their perceptions of group efficacy. Although this may look odd as a result, it makes theoretical sense insofar as we consider that the measure of group efficacy is identity based, and therefore a subject with higher participation in non-normative collective actions would interpret the failure of the movement as a failure of the normative actions carried out by the bogus organization, and thus as a reinforcement of the efficacy of his or her participation in non-normative actions in general.

## General discussion

For the present paper we conducted two experimental studies that sought to determine what is the effect of the perception of success and failure of a social movement in people’s intentions of participating in the future. To accomplish this, we set out three main objectives: to replicate previous findings of the literature regarding the effects of identification with a social movement and having a history of participation; to determine the effects of the outcome of the social movement on perceptions of group efficacy, empowerment and intentions of participating in the future; and to explore the moderating effects of these outcomes on the relationships between social identity and participation on perceived group efficacy, empowerment and intentions participating in collective actions.

First, we found in both studies that current and past participation predict overall participation in the future. Also, interestingly this prediction is action-specific in the sense that a history of participation in normative actions predicts stronger participation in that same type of action; and the same happens with non-normative participation. However, in Study 2 we found an unexpected result regarding the manipulation of participation. Subjects in the participation condition reported having less intentions of keeping participating in the future. We think there may be two explanations for these results. The first one is that people in the non-participant condition actually felt more motivated to keep participating and that reflected in their collective action tendencies measured a week after their exposure to the organization. In this case, it would not be that participants wanted to participate less so much as the non-participants that felt they wanted to do more for the organization. The second possible explanation refers to a concept called “slacktivism.” Some evidence suggests there are some types of low-effort collective actions, such as signing petitions or sharing information online, that can actually decrease a person’s intention of keeping participating in the future (Wilkins et al., 2019). In this context, these low-effort actions may be acting as palliative behaviors that inhibit further engagement in collective action by giving “slacktivists” a sense that they have already done enough.

Also, contrary to our prediction, we found in both studies that current participation has effects on the perception of group efficacy. Similarly, history of participation replicated this effect in Study 1 but not in Study 2. This is not surprising due to the fact that the measure of group efficacy in Study 2 was linked to the bogus organization, and both measures of participation were linked to social movements of interest, there is no reason to believe that having participated previously in other social movements should have an effect on the perception that a “new” organization is effective. Although we did not foresee that participation would have an effect on perceived efficacy, the result does make sense from the DDP perspective given that people that participate have already assessed the movement as potentially able to cope with its goals, and thus, as effective.

Finally, we found that social identity consistently predicted group efficacy, empowerment and participation in normative actions across both studies. These results are consistent with previous literature (van Zomeren et al., 2004, 2008; Drury and Reicher, 2005, 2009).

Regarding the effects of the success and failure of the student movement (*H*_2_), in both studies we found that the perception of success has an impact on group efficacy (although the effect was marginal for Study 2). This is consistent with what was expected through the re-appraisal mechanism described in the DDP model, in which one re-assesses, among other things, the ability of the movement to cope with its goals. In this case, when the question of the social movement’s outcome arises, the re-assessment makes the subjects directly question the efficacy of the movement and theoretically should influence future participation by increasing or decreasing that perception. For instance, a person could interpret failure as a lack of efficacy and therefore reconsider his or her continuity in that movement. This result is also consistent with another study conducted by Tausch and Becker (2013) in which the success and failure of a movement was related to emotional reactions. They found that upon perceiving success the members of a collective action feel pride, as opposed to anger when facing failure. The problem is that theoretically these emotions could trigger either participation or non-participation; and the same happens with efficacy. If the group is perceived as effective and its actions appraised as successful, this could also mean that there is no need to keep participating. To test this dynamic more precisely we looked at potentially moderating effects of the social movement’s outcome on the previously observed effects in *H*_1_.

In Study 1, we found that when facing failure, participants increase their willingness to participate more in the future as opposed to non-participants that actually decrease theirs. We found this pattern predicting intentions of participation in both normative and non-normative collective actions. This would indicate that failure can actually stimulate participants to become more engaged in collective actions. A possible explanation for this finding is that participation is not always associated with effectiveness. Instead, some studies have shown that participants engage in collective actions because they feel a moral obligation to do so, assuming that it is the right thing to do regardless of the effort or risk involved (see Ayanian et al., 2021; Uysal et al., 2022). One study showed that, after failure, participants can increase their moral urgency and commit even harder (Louis et al., 2020). Moreover, our finding about a stronger and more recurrent engagement for non-normative collective actions after failure, implies that when people participate in a collective action through normative means, the failure of the movement can push them into more radical, non-normative behaviors (for similar results see Louis et al., 2020, 2022).

For Study 2, however, the results are different. In this case we found that failure increases the perception of efficacy for those with a history of non-normative participation. When a person has a history of participation in non-normative actions and the social movement faces failure, they could have interpreted that the failure was due to the engagement in the usual normative actions, and hence would increase their perception that the non-normative actions they do are much more effective in accomplishing the goals of the movement. This result is in line with a previous study that found that beliefs in the efficacy of non-normative actions decrease the perceived efficacy of the normative ones and therefore increase the likelihood of participating in non-normative collective actions (Saab et al., 2016).

A number of limitations of this research need to be acknowledged. First, despite the relevance of having a manipulation of participation in our research, the participation condition in Study 2 involved reading and recording an audio message for a campaign, which requires a low level of involvement in comparison to other forms of participation usually seen in the field. Future studies should address this issue by developing ways to manipulate participation that require more involvement or effort from the participants to further differentiate participation from non-participation. The development of social media has expanded the spaces where people can participate in collective actions, from online to offline participation (Chayinska et al., 2021), which opens new opportunities to address this issue. Second, regarding the generalization of our results, it is important to consider that the samples of both studies consisted of Chilean college students, which, although relevant for the social movement we studied, this is not the case in many other collective actions. Future research should consider testing these hypotheses with different populations and in different contexts. Finally, although the statistical power of our studies is reasonable for detecting the univariate effects, the power for detecting the interactions derived from hypothesis 3 is not optimal. Further testing with designs that address this issue are also needed to confirm the findings of this research.

## Data availability statement

The raw data supporting the conclusions of this article will be made available by the authors, without undue reservation.

## Ethics statement

The studies involving human participants were reviewed and approved by Comité Ético Científico en Ciencias Sociales, Artes y Humanidades, Pontificia Universidad Católica de Chile. The patients/participants provided their written informed consent to participate in this study.

## Author contributions

HC, RG, JM, GJ-M, and MCo developed the study idea and designed the study with the support of MCh, CR, BÁ, DV, JD, and AL. MCh, CR, BÁ, and DV implemented the study. CR and MCh carried out the data analysis under the supervision of HC and RG. HC, MCh, and CÁ-D drafted the first version of the manuscript with the support of DC and MV. All authors revised and commented on the manuscript. The final version was approved by all authors.

## Funding

This research was supported by grants from the Chilean National Foundation for Scientific and Technological Development (FONDECYT #1161371), the Center for Social Conflict and Cohesion Studies (ANID/FONDAP #15130009), and the Interdisciplinary Center for Intercultural and Indigenous Studies (ANID/FONDAP #15110006).

## Conflict of interest

The authors declare that the research was conducted in the absence of any commercial or financial relationships that could be construed as a potential conflict of interest.

## Publisher’s note

All claims expressed in this article are solely those of the authors and do not necessarily represent those of their affiliated organizations, or those of the publisher, the editors and the reviewers. Any product that may be evaluated in this article, or claim that may be made by its manufacturer, is not guaranteed or endorsed by the publisher.
